# Integrating bioinformatics and experimental models to investigate the mechanism of the chelidonine-induced mitotic catastrophe via the AKT/FOXO3/FOXM1 axis in breast cancer cells

**DOI:** 10.17305/bb.2023.9665

**Published:** 2024-06-01

**Authors:** Huimin Li, Xiyu Tang, Zhiwei Sun, Zhongyuan Qu, Xiang Zou

**Affiliations:** 1 College of Pharmacy, Harbin University of Commerce, Harbin, China; 2 Pharmaceutical Engineering Technology Research Center, Harbin University of Commerce, Harbin, China

**Keywords:** Chelidonine, breast cancer (BC), AKT/FOXO3/FOXM1 axis, cell cycle arrest, mitotic catastrophe

## Abstract

Breast cancer (BC) is currently the most frequent and lethal cancer among women, and therefore, identification of novel biomarkers and potential anticancer agents for BC is crucial. Chelidonine is one of the main active ingredients of *Chelidonium majus*, which has been applied in Chinese medicine prescriptions to treat cancer. This paper aimed to evaluate the ability of chelidonine to trigger mitotic catastrophe in BC cells and to clarify its mechanism through the AKT/FOXO3/FOXM1 pathway. Bioinformatics analysis revealed that forkhead box O3 (FOXO3) was downregulated in different subtypes of BC. Factors, such as age, stage, Scarff–Bloom–Richardson (SBR) grade, diverse BC subclasses, and triple-negative status, were inversely correlated to FOXO3 levels in BC patients compared with healthy controls. Notably, patients exhibiting higher FOXO3 expression levels demonstrated better overall survival (OS) and relapse-free survival (RFS). Moreover, FOXM1 levels were negatively correlated with both OS and RFS in BC patients. These results revealed that FOXO3 might be considered a predictive biomarker for the prognosis of BC. By utilizing Gene Set Enrichment Analysis (GSEA), we delved into the main Kyoto Encyclopedia of Genes and Genomes (KEGG) enrichment pathways of FOXO3, and the results suggested that FOXO3 was mainly involved in cancer-related pathways and the cell cycle. Thereafter, MTT and flow cytometry (FCM) analysis indicated that chelidonine inhibited BC cell line proliferation and induced M phase arrest. It was found that chelidonine treatment induced MCF-7 cell apoptosis, significantly reduced the expression of survivin and promoted the expression of p53 and caspase-9. Further morphological observation illustrated depolymerization of the actin skeleton and shortening of actin filaments in BC cells, leading to the typical characteristics of mitotic catastrophe, such as abnormal mitosis and multinucleated cells. Western blot analysis demonstrated that chelidonine inhibited the expression of p-AKT to promote the expression of FOXO3 protein and weaken the expression levels of FOXM1 and polo-like kinase 1 (PLK1). Taken together, our present work proved that FOXO3 might be considered a potential therapeutic target for BC. Chelidonine emerges as a promising agent to treat BC by inducing M phase arrest of BC cells and hindering the AKT/FOXO3/FOXM1 axis, thereby inducing mitotic catastrophe in BC.

## Introduction

The most frequently diagnosed cancer and the leading cause of mortality for women globally is breast cancer (BC) [1]. The majority of new tumors in women, approximately 30%, are BCs, according to the most recent epidemiological data [2]. The onset of BC is insidious; early lesions are hard to find and distant metastasis of tumor cells can cause damage and pathological changes in multiple organs. However, as an increasing number of targets and related medications are being developed, there have been great advances in the treatment and prognosis of BC [3, 4].

The convergence point of the complicated oncogenic signaling network is downstream of the cell cycle, and its deregulation is central to the abnormal cell proliferation that characterizes all cancers [5]; therefore, the cell cycle presents a potential target for cancer diagnostics and treatment. Genomic changes throughout the cell cycle cause genomic instability, which is present at different phases of cancer development and is a feature of almost all malignancies [6]. When mitosis is incomplete or fails, a controlled anti-proliferative mechanism known as mitotic catastrophe occurs [7]. Mitotic catastrophe does not constitute a bona fide cell death mechanism in itself, whereas it can be defined as an onco-suppressive mechanism that precedes (and is distinct from) apoptosis, necrosis, or senescence [8]. Avoiding mitotic catastrophe is one of the entry points for the development of cancer. Therefore, the activation of mitotic catastrophe might constitute a highly desirable therapeutic endpoint.

Forkhead box (FOXO) proteins are multifunctional transcription factors that enable the control of gene transcription [9], and they are distinguished by the presence of a conserved forkhead DNA-binding domain [10]. Formerly known as FOXO3a and FKHR-L1 [11], FOXO3 is a member of the class “O” subfamily of FOX proteins (FOXOs). FOXO3 functions downstream of the oncogenic signaling pathway of phosphoinositol-3-kinase (PI3K)-Akt (PKB) and acts as a key player in cell cycle arrest [12], apoptosis, invasion, metabolism, migration, antioxidative stress, and DNA damage [9]. Doxorubicin, a DNA-damaging drug, has been demonstrated to phosphorylate FOXO3, promoting its nuclear localization and activation to regulate cell cycle arrest and apoptosis [13]. Forkhead box protein M1 (FOXM1) is a forkhead protein involved in cell cycle progression, cell differentiation, apoptosis, and DNA damage repair [14, 15]. FOXM1 plays a central role in cancer initiation, and the upregulation of FOXM1 expression is considered to be an early event in cancer development [16]. FOXM1 is one of the most commonly overexpressed genes in different human cancers [17]. Additionally, FOXM1 is the key downstream target of FOXO3, and FOXO3 adversely controls both its expression and activity [18]. Of note, FOXO3 not only directly inhibits the transcription level of FOXM1 and inactivates it but also antagonizes the function of FOXM1 by competing for the same target genes [19]. Briefly, overexpression of FOXO3 leads to cell cycle arrest and apoptosis by inhibiting FOXM1 in most tissues, while its inactivation is beneficial for enhancing cell survival, cell proliferation, and FOXM1 expression. Therefore, targeting the FOXO3–FOXM1 axis may be a viable strategy for cancer treatment.

Chelidonine is one of the main active ingredients of the plant *Chelidonium majus* in the poppy family, which is widespread in Europe and Western Asia. It has been reported that chelidonine has a variety of pharmacological effects, including antitumor [20], anti-inflammatory [21], antiviral [22], antibacterial [23], spasmolytic [24], analgesic [25], and antioxidant [26] effects, among which the antitumor effect has received increasing attention from many researchers. Existing studies have shown that chelidonine could induce SGC-7901 cell mitotic slippage and perish in a manner similar to apoptosis [27], destroy microtubule structures, and induce M phase arrest in lung carcinoma and HeLa cells [28]. Chelidonine also revealed anti-migratory and anti-invasive effects in MDA-MB-231 cells through inhibiting the formation of the ternary IPP complex of integrin-linked kinase, Particularly Interesting New Cysteine-Histidine-rich protein (PINCH), and parvin functions complex and subsequently downregulating IPP downstream signaling molecules, such as AKT and ERK1/2 [29]. In addition, chelidonine induces both apoptosis and autophagy in MCF-7 cells [30] and induces G_2_/M phase arrest and apoptosis in H1975 cells [31]. These studies suggest that chelidonine may act as a potential anticancer agent, while its effects and mechanisms of inducing cell mitotic catastrophe and the relevant signaling pathway have not yet been elucidated.

Herein, we demonstrated through bioinformatics analysis that high FOXO3 expression and low FOXM1 expression were associated with good prognosis in BC patients. Enrichment analysis showed that FOXO3 expression was correlated with the cell cycle and cell division. Subsequently, we experimentally demonstrated that chelidonine caused M phase arrest and mitotic catastrophe in BC cells and eventually induced apoptosis through the AKT/FOXO3/FOXM1 pathway. Our research indicated AKT/FOXO3/FOXM1 as an important pathway in mitotic catastrophe induction and chelidonine as a potential agent to treat BC by intervening in this axis.

## Materials and methods

### Bioinformatics analysis

#### Tumor Immune Estimation Resource (TIMER2.0) database

The *FOXO3* mRNA differential expression level was analyzed in diverse tumor and adjacent normal tissues based on TIMER2.0 (timer.comp-genomics.org/). The Exploration module in TIMER2.0 was used to analyze data, and all data were from The Cancer Genome Atlas (TCGA) database.

#### UALCAN cancer database

The FOXO3 and FOXM1 mRNA and protein differential expression levels were analyzed by the UALCAN cancer database (ualcan.path.uab.edu/). TCGA analysis was used to analyze *FOXO3* and *FOXM1* mRNA expression levels in BC. In addition, we evaluated the protein expression of FOXO3 and FOXM1 by The National Cancer Institute’s Clinical Proteomic Tumor Analysis Consortium (CPTAC) analysis.

#### Kaplan–Meier survival curve analysis

The prognostic significance of FOXO3 and FOXM1 mRNA and protein expression in BC was evaluated in terms of overall survival (OS) and relapse-free survival (RFS) using the Kaplan–Meier plotter (kmplot.com/analysis). To enhance the assessment of the prognostic value of FOXO3 and FOXM1, the patient samples were divided into two cohorts based on auto-select best cut-off for gene expression: high expression and low expression. On the website mentioned above, log-rank *P* values and hazard ratios (HRs) with 95% confidence intervals were calculated.

#### Breast Cancer Gene-Expression Miner v4.9

The correlation between *FOXO3* and *FOXM1* was generated using the correlation module of Breast Cancer Gene-Expression Miner v4.9 (bcgenex.ico.unicancer.fr). The expression module was used to assess the relationship between genes or discovered clusters of associated coexpressed genes.

#### Gene Set Enrichment Analysis (GSEA)

The mRNA expression of 113 normal human breast samples and 1109 cancerous breast samples was obtained from TCGA, from which the *FOXO3* mRNA expression matrix was then extracted. GSEA analysis was carried out to examine the underlying molecular mechanisms between the low-risk and high-risk groups. *P* < 0.05 was considered statistically significant.

#### Functional annotation of coexpressed genes of *FOXO3*

The coexpressed genes of *FOXO3* were screened by using the limma package included in R software [32]. Coexpressed genes were divided into high and low groups based on *FOXO3* expression levels. Using criteria of |logFC| >1 and false discovery rate (FDR) < 0.05, differentially expressed genes were identified by the corresponding R package.

The ClusterProfiler program was used to analyze the Gene Ontology (GO) for molecular function, biological process, and cellular components. A similar approach was adopted for the Kyoto Encyclopedia of Genes and Genomes (KEGG) pathway analysis. FDR and *P* < 0.05 were regarded as significantly enriched.

#### Immunohistochemistry

The FOXO3 immunohistochemistry expression level was assessed in BC patient tumors and normal tissues based on Human Protein Atlas (HPA) databases (www.proteinatlas.org).

#### Molecular docking

The crystal structures of FOXO3 (ID: 7v9b) and FOXM1 (ID: 4yoz) were retrieved from the following website–http://www.pdb.org/–The Research Collaboratory for Structural Bioinformatics Protein Data Bank (RCSB PDB). They were then modified using Autodock tools software. After removing hydrogenated atoms and water molecules, the protein ligand was discarded, but the binding site was retained for further investigation. The chemical structure of chelidonine was downloaded from The Traditional Chinese Medicine Systems Pharmacology (TCMSP) database (http://tcmspnw.com). A flexible docking strategy was then employed to facilitate the interaction between the ligand and receptor showcasing the active pocket at the binding site. Autodock Vina was used for docking, while PyMOL was used to visualize the docking results.

### Experimental cell analysis

#### Chemicals and reagents

Chelidonine (purity: 98%, G30A11C118715) was purchased from Shenzhen Meihe Biological Technology Co., Ltd., while 3-(4,5-dimethylthiazol-2-yl)-2,5-diphenyltetrazolium bromide (MTT, 70080280), RPMI 1640 (MA0215-Oct-06G), DMEM (MA0212-May-28G3), Leibovitz’s L15 (MA0547-Jul-08G), DMEM/F12 (MA0214-Sep-28G), fetal bovine serum (FBS, 21070704), and propidium (P4170) were obtained from Gibco. Vincristine (VCR, purity: 98%, PCS-210402), which was dissolved in RPMI 1640 and stored at −20 ^∘^C, was purchased from Beijing Zhongke Quality Control Biotechnology Co., Ltd. All antibodies were purchased from Beijing Biosynthesis Biotechnology Co., Ltd. Chelidonine was dissolved in DMSO (10 mg/mL) and stored at 4 ^∘^C.

#### Cell source and culture

MCF-7, T47D, MDA-MB-231, MCF-10A, and Bcap37 cell lines were provided by the Cancer Hospital of the Chinese Academy of Medical Sciences. MCF-7 and Bcap37 cells were maintained in RPMI 1640 culture medium, T47D cells were maintained in DMEM, MDA-MB-231 cells were maintained in Leibovitz’s L15 culture medium, and MCF-10A cells were maintained in DMEM/F12 culture medium. All of the media were supplemented with 10% FBS and 1% penicillin–streptomycin. Cells were then cultured in these media at 37 ^∘^C in a humidified incubator with 5% CO_2_. In addition, VCR, a tubulin depolymerizing agent that can inhibit the process of mitotic cell division, was chosen as the positive control.

#### MTT assay

Cells were diluted with RPMI 1640 culture medium containing 10% FBS to a concentration of 3 × 10^4^/mL (MCF-7), 5 × 10^4^/mL (Bcap37, MCF-10A), and 8 × 10^4^/mL (T47D, MDA-MB-231) suspension. Cells (100 µL) were seeded in 96-well culture plates and incubated for 24 h. The following day, the cells were treated with varying concentrations of chelidonine and cultured for 48 h. The media was removed, and the cells were then given 100 µL of medium containing 5 mg/mL MTT and allowed to incubate for 4 h. Afterward, the absorbance at 490 nm was measured in an enzyme mark instrument. Inhibitory concentration (IC_50_) values were calculated relative to the negative control cells.

#### Transmission electron microscopy (TEM) analysis

Cells were diluted to the specified concentrations that follow, 3 × 10^5^/mL (MCF-7), 5 × 10^5^/mL (Bcap37), and 8 × 10^5^/mL (T47D, MDA-MB-231) suspension. Two milliliters of cells were seeded in 6-well culture plates and incubated for 24 h. Then, the cells were treated with 6 µM chelidonine, incubated for 48 h, washed twice with PBS and fixed in ice-cold 2.5% glutaraldehyde (0326A21, Tianjing Alpha Biotechnology Co., Ltd.) in 0.1 M phosphate buffer (pH 7.2) overnight at 4 ^∘^C. The following day, the cells were subjected to three washes with PBS. Subcells were fixed in a 1% aqueous osmium solution, dehydrated using progressively increasing concentrations of ethanol (30%, 50%, 70%, 80%, 90%, and finally, 100%), and finally embedded in araldite. The microtome was implemented to produce ultrathin sections, which were placed on copper grids. The samples were observed by TEM (JEM-1220, Tokyo, Japan) after being stained with 2% aqueous uranyl acetate and lead citrate.

#### Cell cycle assay

Two milliliters of cells (1×10^6^/mL) were seeded in 6-well culture plates and incubated for 24 h. Then, the cells were treated with different concentrations of chelidonine and incubated for 48 h. After centrifuging the cells, the pellet was resuspended in PBS and stained with cell cycle staining solution as per the instructions supplied. Cell cycle analysis was performed using a flow cytometer (CLOUTER EPICS-XL).

#### Colony formation assay

Cells (1×10^4^/well) were plated in 6-well plates for two weeks. After the cells had been fixed with 4% paraformaldehyde, they were stained for 30 min with 0.1% crystal violet and washed in PBS to remove the staining agent, and the colonies were then photographed and counted.

**Figure 1.**
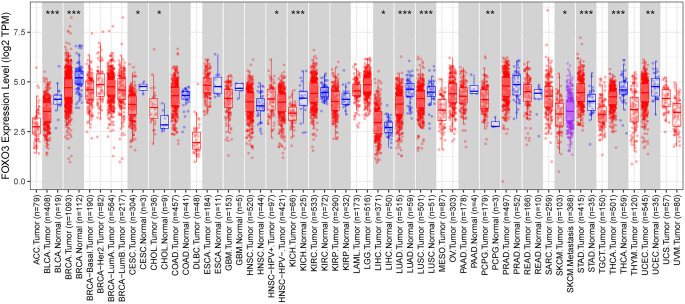
***FOXO3* mRNA expression in diverse tumors.** *P < 0.05, **P < 0.01, ***P < 0.001. FOXO3: Forkhead box O3; BLCA: Bladder urothelial carcinoma; BRCA: Breast invasive carcinoma; KICH: Kidney chromophobe; LUAD: Lung adenocarcinoma; LUSC: Lung squamous cell carcinoma; STAD: Stomach adenocarcinoma; THCA: Thyroid carcinoma.

#### Western blot analysis

After drug treatment, the cells were lysed in cell lysate (prepared with PMSF: cell lysate ═ 1:99) for 15 min on ice. The lysates were centrifuged at 4 ^∘^C and 12,000 rpm for 15 min to collect the supernatants. The total protein content was quantified by a BCA protein assay kit to determine the protein loading volume. Thereafter, 10 µg of each protein sample was subjected to 8%–12% SDS-polyacrylamide gel electrophoresis and transferred to a nitrocellulose membrane (NC). The membrane was cut according to the molecular weight of the target protein, blocked with 5% skim milk at room temperature for 2 h, incubated with the different primary antibodies at 4 ^∘^C overnight and subsequently incubated with the secondary antibodies for 2 h at room temperature. Then, after washing the membrane 3 times with TBST, the immune response band was visualized by using enhanced chemiluminescence (ECL) chromogenic solution on the gel imaging system.

#### Morphological observation

Morphological changes in cells were detected by fluorescence microscopy.

#### Apoptosis assay

MCF-7 cells were seeded in a 6-well plate at 3×10^5^ cells per well for 24 h and treated with 6 µM chelidonine for another 12 h, 24 h, or 48 h. After treatment, the cells were collected and resuspended in 200 µL of 1× Annexin-binding buffer (C1063, Biyuntian Biotechnology Co., Ltd.). The cells were double-stained for 15 min at room temperature in the dark with 5 µL of Annexin-V FITC and 10 µL of PI [33] (C1063, Biyuntian Biotechnology Co., Ltd.). The data were analyzed with a flow cytometer (CLOUTER EPICS-XL).

## Results

### The expression of FOXO3 was negatively correlated with clinical indicators in BC patients

TIMER2.0 analysis demonstrated a significant difference in *FOXO3* mRNA expression between tumor and adjacent normal tissues across several cancers, including bladder urothelial carcinoma (BLCA), breast invasive carcinoma (BRCA), kidney chromophobe (KICH), lung adenocarcinoma (LUAD), lung squamous cell carcinoma (LUSC), stomach adenocarcinoma (STAD), and thyroid carcinoma (THCA) (*P* < 0.05, Figure 1). Specifically, *FOXO3* expression in normal tissues was significantly higher than that in tumor tissues (*P* < 0.05).

**Figure 2.**
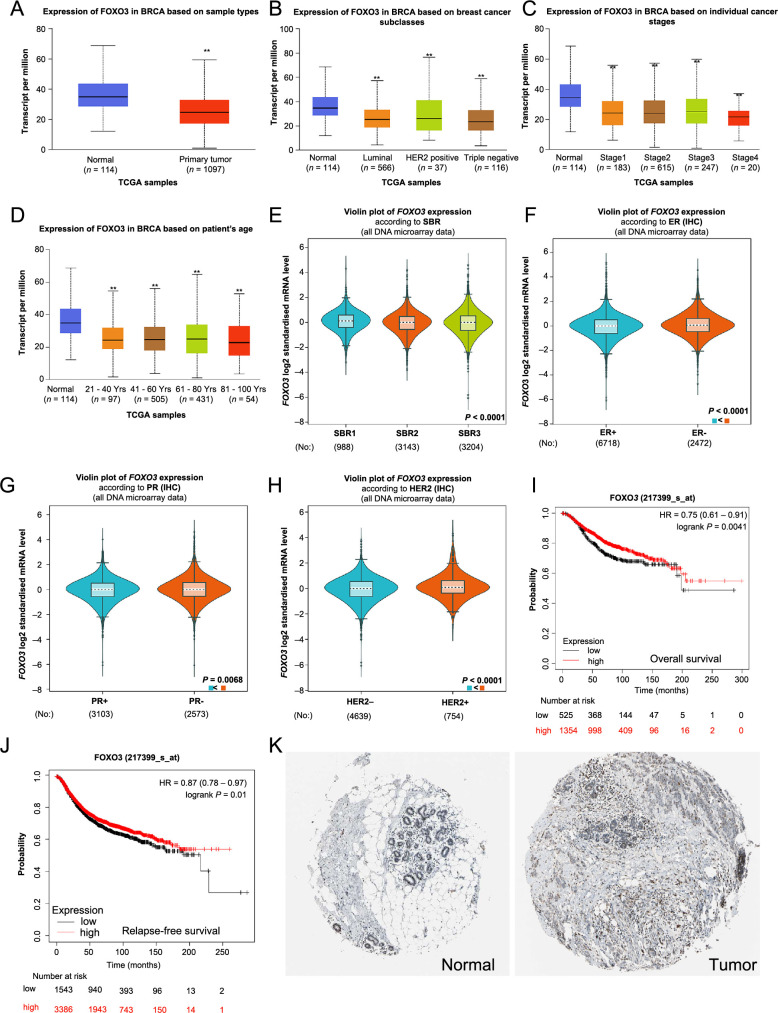
**High expression of FOXO3 was associated with good prognosis in BC patients.** (A–D) *FOXO3* mRNA expression in BC patients by UALCAN cancer database; (E–H) The relationship between *FOXO3* mRNA expression and different clinical indicators using the BC-GenExMiner; (I and J) Kaplan–Meier plotter showing the association between *FOXO3* expression and prognostic; (K) Immunohistochemical data obtained from the HPA database. *P < 0.05, **P < 0.01, ***P < 0.001. FOXO3: Forkhead box O3; BC: Breast cancer; HPA: Human Protein Atlas; UALCAN: The University of ALabama at Birmingham CANcer data analysis portal.

Subsequently, through UALCAN, we evaluated *FOXO3* mRNA expression among groups of patients according to different clinical indicators. These analyses indicated an elevated *FOXO3* expression in normal tissues, while it was reduced in tumor tissues. A marked difference in expression between BC tumor and normal tissues was observed (*P* < 0.01, Figure 2A). In relation to BC major subclasses, the expression of *FOXO3* was significantly lower in luminal, HER2-positive, and triple-negative BC as compared to normal tissue (*P* < 0.01, Figure 2B). We further explored the expression of *FOXO3* based on the individual cancer stage and age of the patients, which revealed its significant reduction across different stages and age groups of BC patients when compared to normal tissue (*P* < 0.01, Figure 2C and 2D).

The Scarff–Bloom–Richardson (SBR) is a histological grading system that evaluates the mitotic index, nuclear pleiomorphism, and tubule development. Patients with higher SBR grades in BC were observed to express diminished *FOXO3* gene expression (Figure 2E). This suggests that reduced expression of *FOXO3* might be linked with poor prognosis in BC patients. In addition, we found that estrogen receptor (ER), progesterone receptor (PR), and HER-2 status were negatively associated with FOXO3 expression (Figure 2F–2H).

Using the Kaplan–Meier plotter, we assessed the prognostic significance of the *FOXO3* gene. Our findings revealed that elevated expression of *FOXO3* correlates with favorable OS (Figure 2I). Conversely, BC patients with downregulated *FOXO3* exhibited poorer RFS (Figure 2J).

The immunohistochemical data of FOXO3, sourced from the HPA database, showcased higher fluorescence intensity in normal tissues compared to tumor tissues (Figure 2K). This observation aligns with the insights obtained from the Kaplan–Meier plotter regarding prognostic value.

**Figure 3.**
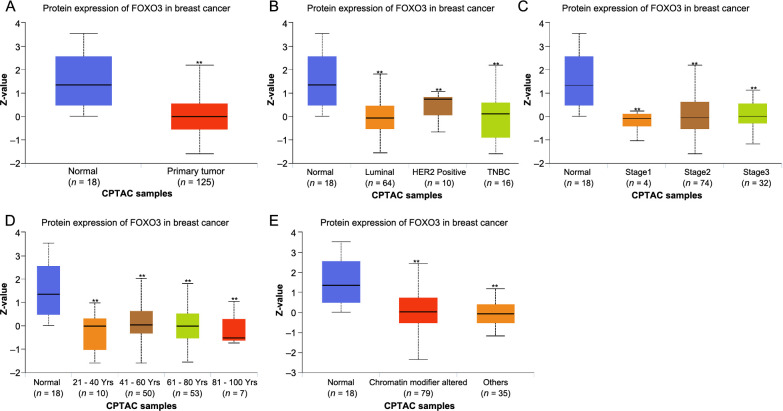
**FOXO3 protein expression in BC patients.** *P < 0.05, **P < 0.01, ***P < 0.001. FOXO3: Forkhead box O3; CPTAC: The National Cancer Institute's Clinical Proteomic Tumor Analysis Consortium; BC: Breast cancer.

The FOXO3 protein expression in BC by CPTAC analysis showed that the FOXO3 protein expression level was consistent with the mRNA expression level. The levels of FOXO3 protein expression were significantly different for major subclasses, individual cancer stages, and ages compared to normal tissue (*P* < 0.01, Figure 3A–3D). Another interesting finding is that FOXO3 protein expression has statistical significance in the chromatin modifier altered compared with normal tissue (*P* < 0.01, Figure 3E), suggesting that FOXO3 may play an important role in cell division.

Therefore, the analysis of FOXO3 mRNA and protein expression revealed a positive correlation between lower *FOXO3* expression and clinical indicators in BC patients. In addition, we found that *FOXO3* expression might be related to mitosis.

### Functional enrichment analysis indicated that *FOXO*3 expression was associated with cancer and the cell cycle

**Figure 4.**
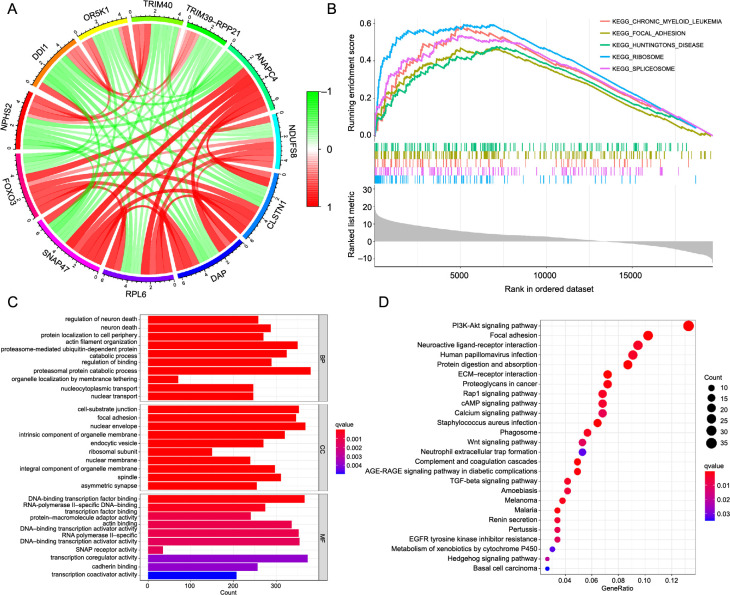
**Functional enrichment analysis.** (A) Co-expression graph; (B) GSEA analysis; (C) GO analysis; (D) KEGG analysis. GSEA: Gene Set Enrichment Analysis; GO: Gene Ontology; KEGG: Kyoto Encyclopedia of Genes and Genomes.

We performed GSEA of the *FOXO3* expression matrix, and the results showed that the concentration was mainly in diverse cancer, cell cycle, immune cell, and cancer-related signaling pathways, including the Notch, MAPK, p53, and Wnt signaling pathways (Figure 4B) (data not displayed). The coexpressed genes of *FOXO3* were screened, and a coexpression graph was formed (Figure 4A). The data demonstrated that FOXO3 had a positive correlation with *ANAPC4*, *DAP*, *CLSTN1*, and *RPL6* and negatively correlated with *DDI1*, *OR5K1*, *TRIM40*, and *TRIM39*-*RPP2o*.

**Figure 5.**
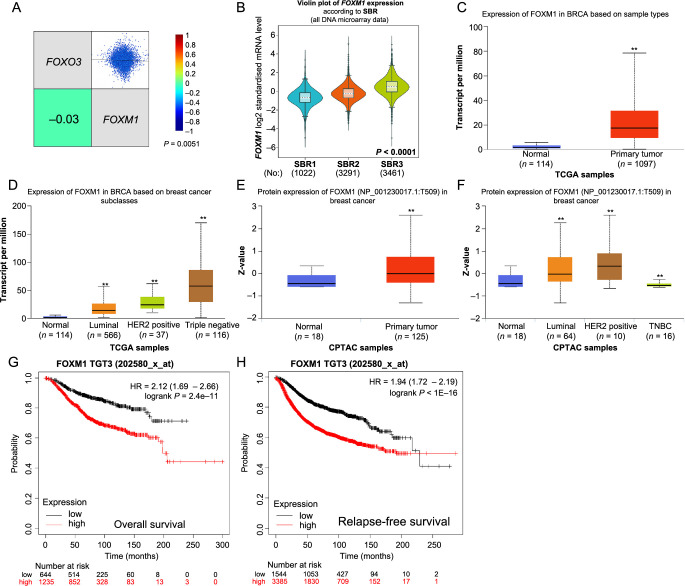
**The mRNA and protein expression of FOXM1 in BC patients and its prognostic through Kaplan–Meier plotter.** (A) Correlation analysis of FOXO3 and FOXM1; (B) SBR; (C and D) *FOXM1* mRNA expression in BC patients; (E and F) FOXM1 protein expression in BC patients; (G and H) Kaplan–Meier plotter showing the association between *FOXM1* mRNA expression and prognostic value. FOXM1: Forkhead box protein M1; BC: Breast cancer; FOXO3: Forkhead box O3; SBR: Scarff–Bloom–Richardson; TNBC: Triple-negative breast cancer.

To further verify the function of FOXO3, we performed functional enrichment analysis of its coexpressed genes (Figure 4C and 4D). The results of biological process analyses showed that the coexpressed genes of *FOXO3* were involved in neuron death, actin filament organization, and intracellular transport. Cellular components analysis highlighted associations with the nuclear membrane and spindle. Molecular function analysis indicated that the coexpressed genes of *FOXO3* were primarily located in DNA-binding transcription and transcription coregulator activity. In the KEGG pathway analyses, these genes were mainly involved in various cancers and cancer-related signaling pathways.

Conclusively, this functional enrichment analysis indicates FOXO3’s pivotal role in diverse cancer and cancer-related signaling pathways. We found that FOXO3 expression was associated with the cell cycle and cell division.

### Increased expression of FOXM1 was correlated with poor prognosis in BC patients

It was reported that FOXM1 interacted with FOXO3, based on previous studies [34]. We also verified the negative correlation of *FOXO3* and *FOXM1* by Breast Cancer Gene-Expression Miner v4.9 (*P* < 0.01, Figure 5A). In addition, BC patients with a more advanced SBR grade tend to express higher FOXM1 (Figure 5B), leading us to infer that elevated FOXM1 expression might correlate with poor prognosis of BC patients.

We found that FOXM1 mRNA and protein were highly expressed in tumor tissue with the same trend (Figure 5C and 5E). Regarding BC major subclasses, the expression of FOXM1 was significantly higher in luminal, HER2-positive, and triple-negative BC tissues than in normal tissues (Figure 5D and 5F). Kaplan–Meier plotter was applied to predict the relationship between FOXM1 and prognosis in BC patients. The data analysis showed that a lower expression of FOXM1 was associated with a better prognostic value, which was consistent with the SBR analysis.

### Chelidonine had good binding activity with FOXO3 and FOXM1

**Figure 6.**
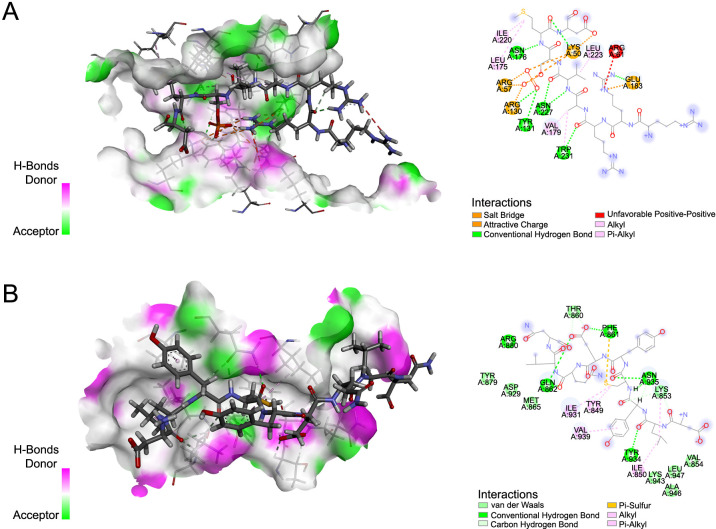
**Chelidonine had good binding activity with FOXO3 and FOXM1.** (A) Protein-molecule docking of FOXO3 and chelidonine; (B) Protein-molecule docking of FOXM1 and chelidonine. FOXO3: Forkhead box O3; FOXM1: Forkhead box protein M1.

To further verify whether chelidonine can treat BC through FOXO3 and FOXM1, we predicted the interaction between them through molecular docking experiments. Figure 6 illustrates the docking results, wherein the green segment denotes hydrogen bonding interactions. Notably, the docking of FOXO3 with chelidonine revealed five hydrophobic bonds and eight classical hydrogen bonds, which are strong hydrogen bonds formed by the residue O atom with the ligand H atom. The results of FOXM1 docking with chelidonine showed that five classical hydrogen bonds and five hydrophobic bonds were formed. Furthermore, the binding activity was predicted based on the binding energy in kcal/mol. A binding energy lower than −7.0 kcal/mol indicated strong binding activity [35]. Chelidonine had a binding energy of −7.3 kcal/mol with FOXO3 and −7.8 kcal/mol with FOXM1, which provides further support for chelidonine having a good binding effect with FOXO3 and FOXM1. Therefore, we believe that chelidonine may play a therapeutic role through the FOXO3/FOXM1 axis.

### Chelidonine impaired BC cell proliferation and caused apoptosis

**Figure 7.**
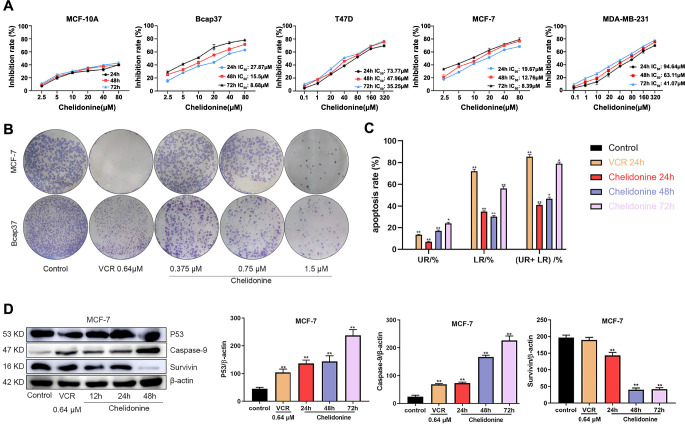
**Chelidonine impaired BC cells proliferation and caused apoptosis.** (A) The anti-proliferative effect of chelidonine on BC cells by MTT assay; (B) Colony formation of MCF-7 and Bcap37 treated with chelidonine; (C) Apoptosis analysis MCF-7 cells treatment with 6 µM chelidonine at different time using FCM, UR: late apoptosis, LR: early apoptosis, (UR+ LR): total apoptosis; (D) Western blotting analysis of apoptosis-related proteins protein p53, caspase-9, and survivin of MCF-7 after 6 µM chelidonine treatment at different time point; All samples derive from the same experiment and that blots were processed in parallel. *P < 0.05, **P < 0.01, ***P < 0.001. BC: Breast cancer; FCM: Flow cytometry.

The MTT assay was first applied to determine the antiproliferative effect of chelidonine on BC cell lines. Results demonstrated that chelidonine treatment for 24, 48, and 72 h impaired cell proliferation in MCF-7, Bcap37, T47D, and MDA-MB-231 cells (Figure 7A), with IC_50_ values summarized in Table 1. Interestingly, the data showed that MCF-7 and Bcap37 cells were more sensitive to chelidonine. Meanwhile, chelidonine exhibited low toxicity to MCF-10A breast epithelial cells within the experimental dose range. Then, colony formation analysis was carried out, and the results showed that colony formation of MCF-7 and Bcap37 cells was remarkably suppressed in a dose-dependent manner (Figure 7B). Therefore, these findings suggest that chelidonine may specifically inhibit the proliferation of MCF-7 and Bcap37 cells, with MCF-7 cells showing pronounced sensitivity to concentrations of 0.64 µM VCR and 1.5 µM chelidonine.

To further explore the mode of cell death of BC cells, we examined the apoptosis induced by 6 µM chelidonine in MCF-7 cells. Chelidonine and VCR therapy greatly increased the percentage of apoptotic cells (*P* < 0.01, Figure 7C), although the effect was not time dependent. In addition, the activation of p53, caspase-9, and survivin, the standard indicators of apoptosis, was also detected (Figure 7D). Under a variety of cellular stressors, the p53 tumor suppressor can activate a wide range of downstream target genes that are in charge of p53-dependent cell cycle arrest or apoptosis [36]. After chelidonine treatment, p53 protein expression increased in a dose-dependent manner (*P* < 0.01). The initiator caspases (caspase-2, 8, 9, and 10) are assembled into multiprotein complexes, such as PIDDosomes, DISCs, or apoptosomes during the induction of apoptosis, which is one of the main functions of caspases [37, 38]. Survivin, a member of the family of proteins known as inhibitors of apoptosis, is abundantly expressed in a variety of cancers and performs critical functions in cell survival and proliferation [39]. The results showed that 6 µM chelidonine and VCR significantly increased caspase-9, while the pro-survival regulator survivin was suppressed in a time-dependent manner.

**Table 1:** IC_50_ values of chelidonine on different breast cancer cell lines

**Cell lines**	**IC_50_ (µmol/L)**	
	**24 h**	**48 h**	**72 h**
MCF-7	19.6 ± 0.89	12.80 ± 0.73	8.41 ± 0.53
Bcap37	27.87 ± 1.77	15.55 ± 0.84	8.69 ± 0.62
T47D	73.97 ± 3.92	47.97 ± 0.58	35.27 ± 0.53
MDA-MB-231	94.64 ± 2.65	63.09 ± 4.52	41.07 ± 0.83

IC_50_: Half-maximal inhibitory concentration.

### Chelidonine-induced M phase arrest and mitotic catastrophe in BC cells

**Figure 8.**
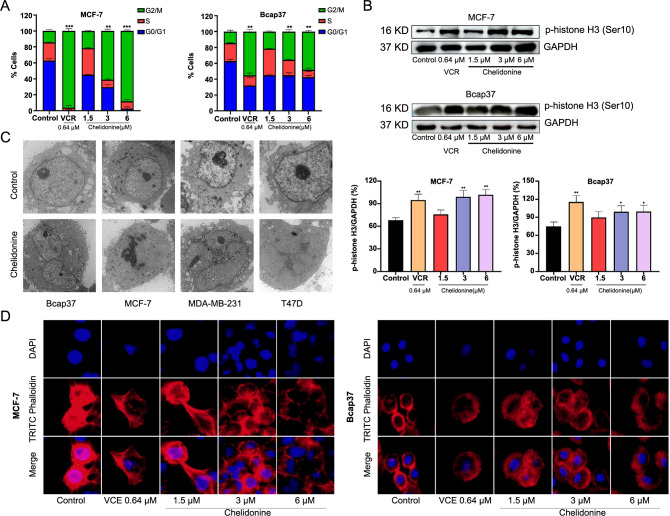
**Chelidonine-induced M phase arrest and mitotic catastrophe in MCF-7 and Bcap37 cells.** (A) Cell cycle analysis; (B) Western blotting analysis of M phase marker protein, all samples derive from the same experiment and that blots were processed in parallel; (C) The morphology of BC cells was detected by TEM, scale bar: 10 µm; (D) The actin cytoskeleton changes of MCF-7 and Bcap37 cells after treatment with chelidonine, scale bar: 50 µm. *P < 0.05, **P < 0.01, ***P < 0.001. TEM: Transmission electron microscopy; BC: Breast cancer.

Moreover, the effect of chelidonine on the morphology of BC cells was observed by TEM. The standard morphological hallmarks of tumor cells, such as obvious cellularity, integrated organelle structures, and uniform chromatin distribution, were present in the untreated MCF-7, Bcap37, T47D, and MDA-MB-231 cells. After treatment with 6 µM chelidonine for 48 h, the volume of each cell line increased significantly, cells displayed an irregular shape. Nuclear membranes were formed randomly around the chromatin, resulting in the emergence of multiple micronuclei and the formation of multinucleated cells, which are the main phenomena of mitotic catastrophe [7] (Figure 8C). Among them, MCF-7, T47D and MDA-MB-231 cells exhibited pronounced proliferation during the nuclear fission phase. However, MDA-MB-231 cells also displayed noticeable chromatin condensation and nuclear fragmentation, indicative of typical apoptotic characteristics.

**Figure 9.**
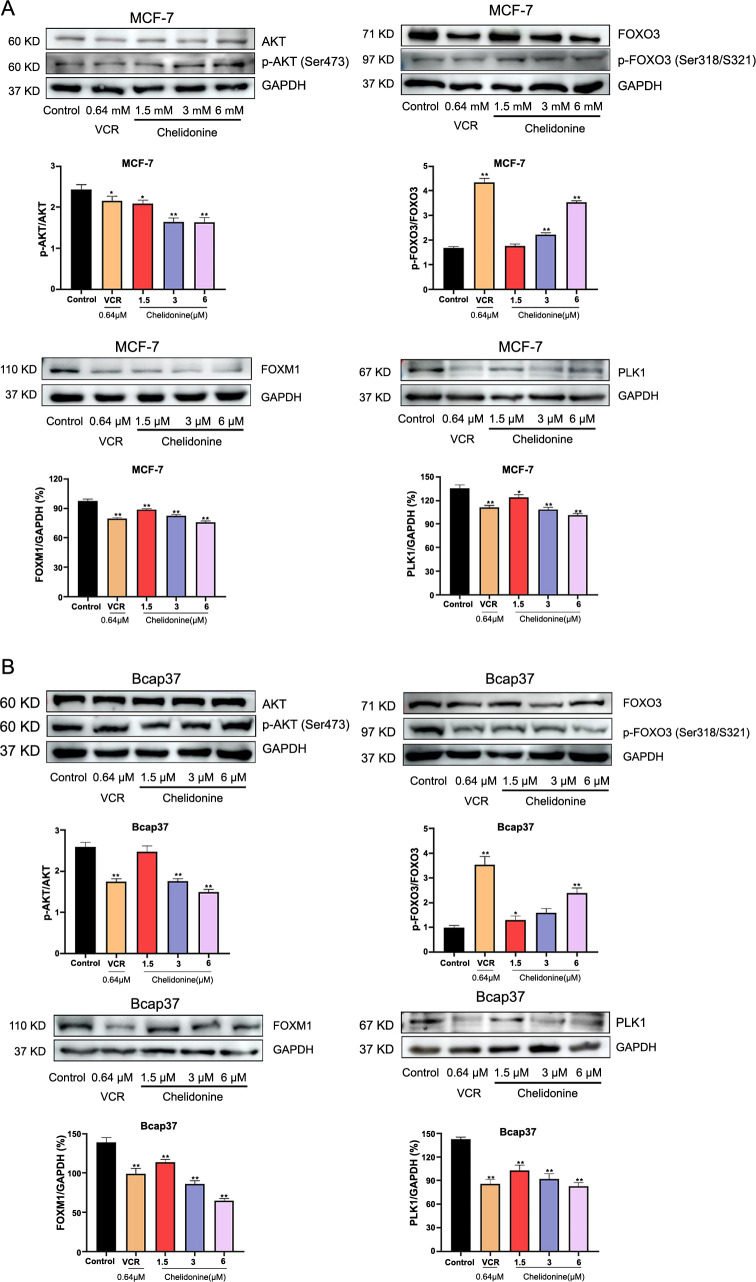
**Chelidonine activated the AKT/FOXO3/FOXM1 axis.** Chelidonine activated the FOXO3 and expression of downstream proteins FOXM1 and PLK1 expression in MCF-7 (A) and Bcap37 cells (B); All samples derive from the same experiment and those blots were processed in parallel. *P < 0.05, **P < 0.01, ***P < 0.001. FOXM1: Forkhead box protein M1; PLK1: Polo-like kinase 1.

The anticancer properties of chelidonine were further clarified by evaluating its impact on cell cycle distribution with flow cytometry (FCM). Following a 48-h treatment with concentrations of 3 and 6 µM chelidonine, a significant increase in the G_2_/M phase ratio was observed in MCF-7 and Bcap37 cells (*P* < 0.01, Figure 8A). Given that mitotic chromatin condensation and histone H3 (Ser-10) phosphorylation are correlated, it serves as a marker for cells transitioning from the G_2_ phase into the M phase [40, 41]. Therefore, western blot analysis was conducted to examine the expression of p-histone-H3, and a significant increase in p-histone-H3 expression in MCF-7 and Bcap37 cells was observed after treatment with 3 and 6 µM chelidonine (Figure 8B), suggesting that chelidonine induced M phase arrest in BC cells.

The actin cytoskeleton is a key component of eukaryotic cells and plays an important role in centrosome separation, spindle assembly, and chromosome localization, among which f-actin surrounds the chromosomes, presents throughout the entire cytoplasm during mitosis, forms a contraction ring and participates in cytokinesis [42, 43]. To further confirm whether chelidonine caused mitotic catastrophe in BC cells, we observed changes in the actin cytoskeleton by fluorescence microscope. With increasing chelidonine concentration, the actin skeleton of MCF-7 and Bcap37 cells underwent drastic changes and remodeling. Cytoplasmic division failed, and multinucleated cells appeared, leading to mitotic catastrophe in MCF-7 and Bcap37 cells (Figure 8D). These findings were consistent with the TEM observations (Figure 8C), which suggested that chelidonine could induce mitotic catastrophe in BC cells.

### AKT/FOXO3/FOXM1 pathway involved in chelidonine-induced mitotic catastrophe

Functional enrichment analysis indicated that FOXO3 was mainly involved in actin biological processes and the AKT signaling pathway, leading us to assess the effect of chelidonine on the AKT/FOXO3/FOXM1 signaling pathway. Previous reports have shown that FOXO3 and FOXM1 function downstream of the PI3K-AKT signaling cascade and are crucial for cell proliferation and cell cycle control [34]. Therefore, western blotting was applied to analyze the expression of AKT/FOXO3/FOXM1 signaling pathway-related proteins after chelidonine treatment for 12 h in MCF-7 and Bcap37 cell lines. Compared with the control group, phosphorylation of AKT (p-AKT) levels were significantly reduced after chelidonine treatment (*P* < 0.05, Figure 9). Meanwhile, we noticed that chelidonine treatment dramatically increased the activity of p-FOXO3 in a dose-dependent manner (*P* < 0.05, Figure 9). Subsequently, we detected the expression of the FOXO3 downstream proteins FOXM1 and PLK1. We found that chelidonine treatment decreased the expression of FOXM1 and PLK1 in a dose-dependent manner (*P* < 0.05, Figure 9). Thus, the AKT/FOXO3/FOXM1 signaling pathway could be a novel target for the treatment of BC with chelidonine.

## Discussion

Mitotic catastrophe results from cells entering mitosis improperly, or it can occur immediately after dysregulated or unsuccessful mitosis [8]. There is usually some level of mitotic arrest present during mitotic catastrophe. Chromosome breakage and poor karyokinesis, which result in nuclear changes (micronucleation and multinucleation) that are the most noticeable morphological characteristics of mitotic catastrophe, are frequently associated with failing mitoses [8]. However, cytoplasmic shrinkage (also known as pyknosis), chromatin condensation, permeabilization of the mitochondrial membrane, and activation of caspase can also be observed in cells that have failed mitosis [6, 44]. This result suggested that mitotic catastrophe senses mitotic failure and responds to cell death by driving apoptosis. In this study, we first found that chelidonine inhibited the proliferation of different BC cell lines and exhibited low toxicity to normal cells. Further studies showed that chelidonine mediated M phase arrest and apoptosis of BC cells. Chelidonine treatment induced BC cell apoptosis, significantly reduced the expression of survivin, and promoted the expression of p53 and caspase-9. Our results are consistent with the previously reported chelidonine-induced apoptosis of MCF-7 [30]. In addition, morphological observation suggested that chelidonine treatment could depolymerize the actin skeleton and shorten the actin filaments of BC cells, leading to the typical characteristics of mitotic catastrophes, such as abnormal mitosis and multinucleated cells [7]. This is consistent with previous reports that chelidonine can affect the cytoskeleton of tumor cells and block them in the G2/M phase [45]. Therefore, we speculated that chelidonine-induced mitotic catastrophes in BC cells and subsequently promoted apoptosis.

It is still unclear which molecular signaling mechanisms cause mitotic catastrophe. Strong evidence exists that FOXO3, which acts as a tumor inhibitor, regulates cell cycle arrest, cell death, and DNA damage repair [46]. It is becoming obvious that FOXO3 must be active to keep cells under control and that FOXO3 inactivation is linked to features of cancer cells [14, 47]. According to the analysis of bioinformatics results, it might be possible to predict the prognosis of BC using the expression of FOXO3. Using Kaplan–Meier plotter, we found that BC patients with higher expression of FOXO3 had improved OS and RFS. To further explore the role of FOXO3 in BC, GSEA and functional enrichment analyses were performed. Interestingly, the results showed that FOXO3 was mainly expressed in cancer and cancer-related pathways, such as the PI3K-AKT pathway, and its main biological function was involved in the cell cycle [48], which was consistent with the results of SBR. These findings suggested that FOXO3 expression may serve as a biomarker for predicting BC prognosis.

Mitotic catastrophe occurs when the mitotic process is disrupted or DNA is directly damaged [7]. A major player in the regulation of the DNA damage response (DDR), FOXO3a largely controls DNA repair, cell cycle checkpoint arrest, and the propagation of DDR signals [49]. In the presence of various intracellular and extracellular stimuli, the multiple cellular proliferative signaling pathways were mainly concentrated on FOXO3 [34]. In addition, FOXO3 regulates the proteins that affect the cell cycle and proliferative arrest. In the G2/M phase, overexpression inhibiting their expression is FOXO3a's binding to the cyclin B1 and Polo-like kinase 1 (PLK1) promoter regions [50]. PLK1 participates in mitotic spindle formation, centrosome maturation, chromosomal segregation, and cytokinesis during the course of the mitotic cycle [51, 52]. Supernumerary centrosomes arise from deregulation of the centrosome cycle, leading to multipolar mitosis, this in turn promotes genomic instability and mitotic catastrophe [51, 52]. Moreover, PLK1 is a binding partner and a negative regulator of FOXO3 tumor suppressor [53]. Overexpression of FOXO3 enhances DNA damage repair and the S-phase and G2/M cell cycle checkpoints, while FOXO3 deletion impairs these DDR processes [18]. These reports were consistent with our predictions from the database that FOXO3 expression was negatively correlated with SBR, which is involved in the regulation of the cell cycle. We found that chelidonine blocked the M phase of BC cells with low FOXO3 expression and inhibited the expression of PLK1. Most crucially, in response to a range of external stimuli and growth factors, PI3K-AKT signaling pathways adversely control FOXO3 in a number of ways [54]. The PI3K-AKT pathway is activated upstream of FOXO3, and once the process is active, AKT phosphorylates FOXO3 to create binding sites for the chaperone protein 14-3-3 in the nucleus. This in turn controls FOXO3 transcriptional activity and cytoplasmic translocation [54, 55]. Western blotting showed that chelidonine inhibited the activity of AKT and increased the expression of FOXO3. The results of western blotting are consistent with previous reports that chelidonine can inhibit the activation of AKT in BC cells [29]. Therefore, we speculated that chelidonine might promote FOXO3 expression by inhibiting p-AKT expression, thereby promoting BC cell G/M phase arrest and mitotic catastrophe.

Many malignancies are characterized by the expression of FOXM1, and it has been suggested that this upregulation of FOXM1 expression occurs early in the development of cancer. In the database study, we discovered that increased FOXM1 expression was linked to a poor prognosis in BC patients, suggesting that it may be a biomarker for predicting BC prognosis. FOXM1 is an important constituent in the regulation of G1/S and G2/M cell cycle transitions, maintenance of mitotic spindle integrity, DNA damage repair, and apoptosis, and the absence of any of these functions contributes to the development of tumors [56]. After activation by the mitotic kinase PLK1, FOXM1 levels peak in the G1/S and G2/M stages [56]. FOXM1 causes anomalies in the cell cycle, such as prolonged G2/M processes, chromosome mis-segregation, and failure of cytokinesis, when it is deleted [57]. Genome instability and mitotic catastrophe are caused by chromosomal mis-segregation and cytokinesis failure in the following cell cycle [58, 59]. The above report was similar to our findings based on the database, where FOXM1 was positively correlated with SBR.

One of FOXO3’s most significant and crucial downstream transcriptional targets, particularly in terms of the control of the DDR, is FOXM1 [49]. FOXM1 has been found to be overexpressed in cancer cells that are resistant to drugs that damage their DNA, and its expression can block the effectiveness of genotoxic agents [60]. Additionally, FOXM1 is essential for both drug responsiveness and resistance [14]. For example, paclitaxel mediates mitotic mutation and senescence in BC by downregulating FOXM1 [61], and upon treatment with epirubicin via an E2F-element on its promoter, p53 has been demonstrated to inhibit the transcription of FOXM1 in BC cells [62]. Downregulation of FOXM1 expression promotes DNA damage-induced cancer cell death and disrupts mitosis/cytokinesis [62, 63]. In this study, we demonstrated that chelidonine treatment reduced FOXM1 and PLK1 expression, which implied that chelidonine may inhibit proliferation of BC cells by inhibiting the AKT/FOXO3/FOXM1 pathway.

Overall, these results imply that targeting the FOXO3/FOXM1 axis is a conceptually sound therapeutic strategy for the treatment of BC due to the transcriptional regulation of genes involved in cell cycle control and DDR by the FOXO3/FOXM1 axis, as well as the possibility that it is linked to mitotic catastrophe. However, the role of the FOXO3/FOXM1 axis in mitotic catastrophe needs to be further explored.

## Conclusion

Our findings in the present study indicate that FOXO3 was downregulated in different subtypes of BC. Age, SBR grade, diverse BC subclasses, and triple-negative status were negatively related to FOXO3 levels in BC samples compared with normal tissues. Patients with increased FOXO3 expression levels showed better OS and RFS. It might be implied that FOXO3 might be a predictive biomarker for the prognosis of BC. In addition, we found that chelidonine suppressed cell proliferation and induced M phase arrest and mitotic catastrophe in BC cells by regulating the AKT/FOXO3/FOXM1 axis. This work paved the way by being the first to establish the relationship between FOXO3 and FOXM1 in chelidonine stimulation and presented a different pathway for additional clinical research in BC.
